# THE FAMILY-CENTERED FUNCTION-FOCUSED CARE INTERVENTION: IMPLEMENTATION AND MEASUREMENT CONSIDERATIONS

**DOI:** 10.1093/geroni/igac059.380

**Published:** 2022-12-20

**Authors:** Marie Boltz, Jane Flanagan

## Abstract

Family care partners can play an important role in promoting the functional recovery of hospitalized older adults living with dementia. Family-centered function-focused care (Fam-FFC) aims to create an “enabling” milieu for the person with dementia through, staff education, unit-based champions, environmental and policy assessment/modification, and individualized goal setting. In this patient/family-centered care approach, nurses purposefully engage family care partners in the assessment, decision-making, care delivery and evaluation of function-focused care during hospitalization and the 60-day post-acute period. Staff are provided with techniques to support positive interaction with patients and families and respect the role of care partners as advocates. The goal is to promote functional recovery and well-being in the patient, and prevent unnecessary long-term care admissions, while improving care partners’ preparation for caregiving and well-being. Fam-FFC was developed with the input of patients, families, nurses, and rehab therapists. This symposium focuses on key factors associated with the implementation of Fam-FFC, and relevant measures. After an introductory overview of Fam-FFC, the nature of staff/patient interaction during hospitalization, including the influence of gender, will be discussed in the first presentation. Two presentations will address the planning for transition to the post- acute setting, including the intrinsic factors associated with preparation for caregiving, in contrast to the decision to seek long-term care. The final presentation will describe the approach to promoting fidelity to the Fam-FFC intervention, within the context imposed by COVID-19 restrictions. Our symposium will conclude with a discussion of future direction for policy, practice, and research.

